# P02.38. Effectiveness of reflexology in improving perioperative patient centered outcomes: a comparative effectiveness study

**DOI:** 10.1186/1472-6882-12-S1-P94

**Published:** 2012-06-12

**Authors:** S Attias, E Schiff

**Affiliations:** 1 Bnai Zion Medical Center, Haifa, Israel

## Purpose

Perioperative symptoms such as pain and anxiety are common in spite of standard of care. Such symptoms are associated with a negative surgery experience, and moreover, are correlated with increased perioperative morbidity. The aims of this study were to evaluate whether reflexology as an add-on to standard of care improves these symptoms. In addition, we assessed whether outcomes are correlated with expectations from CAM.

## Methods

We conducted a pragmatic trial of 234 adult patients undergoing various abdominal operations. 89 patients received standard medical care, and 145 patients received reflexology on top of standard medical care, according to patient preference and practitioner availability. Numeric VAS scores for anxiety, pain, and well-being were collected pre and post treatment.

## Results

There was a significant reduction of VAS scores for all outcomes in the reflexology group: Anxiety scores were reduced from 5.2 to 2.2 (n=145, p<0.0001), pain from 5.3 to 2.9 (n=79, p<0.0001), and well being improved from 5.2 to 6.7 (n=69, p<0.0001). Symptomatic improvement was significantly better in the reflexology group as compared to the standard of care group for all parameters (p>0.0001). In the subgroup of patients experiencing moderate to severe symptoms, improvement was even more prominent: Anxiety scores were reduced from 7.1 to 2.7 (n=94, p<0.0001); pain from 7.2 to 4 (n=55, p<0.0001); and well being improved from 3.7 to 6.4 (n=47, p<0.0001). We did not find a correlation between outcomes and patients’ expectations regarding reflexology.

## Conclusion

Our results demonstrate that reflexology therapy significantly improved common symptoms in patients undergoing surgical interventions.

